# Intracerebral hemorrhage complicating viral hepatitis A

**DOI:** 10.11604/pamj.2015.22.191.3973

**Published:** 2015-10-26

**Authors:** Hatim Belfquih, Brahim Elmostarchid

**Affiliations:** 1 Department of Neurosurgery, Mohammed V Military Teaching Hospital, Rabat, Morocco

**Keywords:** Intracerebral hemorrhage, viral hepatitis A, anti-HVA IgM, complicated

## Image in medicine

The 11-year old girl presented a diagnosis of viral hepatitis A confirmed by anti-HVA IgM. Eight 8 weeks after, she developed hemorrhagic syndrome coupled with fever associated and persistent jaundice. The blood count showed aregenerative pancytopenia secondary to aplastic anemia confirmed by bone marrow biopsy. During conditioning for allogenic bone marrow grafts, the patient developed generalized seizures revealing disseminated intracerebral hemorrhage (pictures). There was a favorable outcome after blood transfusions and resuscitation. Hematological complications in hepatitis A are rare. Although intracerebral hemorrhage resulting from Hep A has never been reported in the literature; its occurrence is directly correlated with the degree of thrombocytopenia due to aplastic anemia. The prevalence of aplastic anemia in viral hepatitis is estimated at 0.1%. This complication implies systematic hematological evaluation in cases of viral hepatitis A; especially in severe forms or those associated with involving cholestasis.

**Figure 1:**
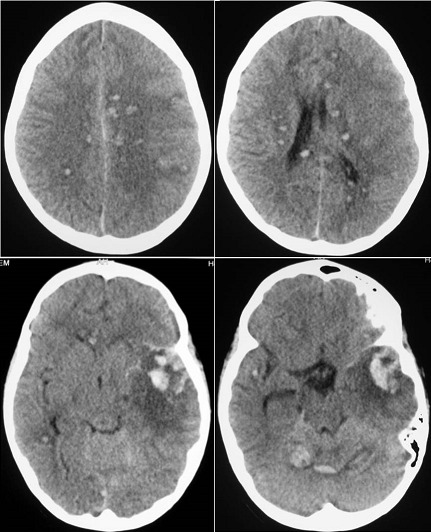
Cranial computed tomography scan showing disseminated intracerebral hemorrhage

